# Nitrogen-Doped Hierarchically Porous Carbons Derived from Polybenzoxazine for Enhanced Supercapacitor Performance

**DOI:** 10.3390/nano9010131

**Published:** 2019-01-21

**Authors:** Yanhui Wang, Liyan Dong, Guiping Lai, Meng Wei, Xingbi Jiang, Lizhong Bai

**Affiliations:** Department of Materials Science and Engineering, North University of China, Taiyuan 030051, China; yanhuiwang9@gmail.com (Y.W.); 18834160076@139.com (L.D.); 18406581311@sina.cn (G.L.); 18834165330@139.com (M.W.); 15735658082@163.com (X.J.)

**Keywords:** supercapacitor, polybenzoxazine, hierarchically porous carbons, nitrogen-doped

## Abstract

Nitrogen-doped hierarchically porous carbons (HPCs), which are synthesized from benzoxazine resins, were successfully prepared following the processes of polymerization, carbonization, and potassium hydroxide (KOH) activation. As the key factor, the KOH activation temperature influences the pore structure and surface functionality, which are crucial for the excellent performance. The HPC-800 material, with the highest activation temperature (800 °C), displays a hierarchical pore structure, a high specific surface area (1812.4 m^2^·g^−1^), large total pore volume (0.98 cm^3^·g^−1^), high nitrogen content (1.27%), and remarkable electrical conductivity. It has also presented an excellent electrochemical performance of high specific capacitance of 402.4 F·g^−1^ at 0.1 A·g^−1^, excellent rate capability of 248.6 F·g^−1^ at 10 A·g^−1^, and long-term cycling stability with >99.0% capacitance retention after 500 cycles at 1 A·g^−1^ in 6 M KOH aqueous solution.

## 1. Introduction

As a new kind of advanced energy storage device, supercapacitors have attracted more research interest, and have been used in electronic devices, electric vehicles, and renewable power systems [1,2]. Generally, supercapacitors perform higher energy densities than conventional capacitors and deliver better power density than rechargeable lithium ion batteries [3,4]. Due to their large specific surface area, high electric conductivity, excellent chemical stability, and good capacitive performance [5,6,7], nanoporous carbons have recently been widely used as electrode materials for supercapacitors. The capacitive performance of nanoporous carbon electrodes remarkably depend on their textural and structural characteristics [8,9], such as specific size distribution and degree of graphite crystallinity, etc.

Hierarchically nanoporous carbons (HPCs) performing a multimodal pore size distribution of micro-, meso-, and/or macropores, can reduce the diffusion distance and in-pore ion-transport resistance, and an enhanced electrochemical capacitance can be obtained [10]. As a nitrogen functionality in the carbon framework, the nitrogen-doped HPCs can supply a reversible pseudo-capacitance from Faradaic electrochemical reactions at the electrolyte/electrode interfaces, and enhance the electronic conductivity and surface wettability. Therefore, most nitrogen-doped HPCs are considered an ideal electrode material for high-performance supercapacitors [11]. Currently, various carbon precursors have been successfully converted into nitrogen-doped HPCs, such as polyacrylonitrile, polyaniline, phenolic resin, biomass, and biomass derivatives [12].

Polybenzoxazines (PBZs), a new type of heterocyclic polymers, are derived from ring-opening polymerization of benzoxazine monomers [13]. Benzoxazine monomers have been readily synthesized from phenols, formaldehyde, and amines by Mannich condensation [14]. Polybenzoxazines become a promising candidate for high-performance nitrogen-doped HPCs [15], because of molecular design flexibility, high char yield, good thermal stability, and hardly any shrinkage. Furthermore, the content and style of nitrogen species can be easily adjusted by changing the sot or proportion of amines in PBZs [16]. However, until now, there are few reports on the nitrogen-doped HPCs derived from PBZs for supercapacitors. Wan et al. [17] reported the nitrogen-doped porous carbons (NPCs) prepared from a novel nitrile-functionalized benzoxazine using soft-templating and KOH activation method, which possess an abundant micro/mesoporous structure, large surface area, high nitrogen and oxygen content, and excellent electrical conductivity. The NPCs were applied as electrode materials for supercapacitors, and the NPC-2 sample activated at 700 °C exhibited the highest specific capacitance of 362.4 F·g^−1^ at 1 A·g^−1^, and the capacitance retention of 94.7% after 5000 cycles. 

In this paper, we synthesize a group of novel nitrogen-doped HPCs with the hierarchical pore structure derived from the PBZ, after then KOH chemical activated at different temperatures. The PBZ was prepared from phonel, aniline, and paraformaldehyde via a solventless method and then thermally ring-opening polymerization. The pore structure and surface functionality of HPCs can be tailored by changing the activation temperature. To ascertain the potential for supercapacitor electrode materials, the HPCs were investigated, and the electrochemical capacitive behavior in three- and two-electrode systems with 6 M KOH aqueous electrolyte. In addition, the influence of pore structure and surface nitrogen functional groups of the HPCs on electrochemical capacitive performance are discussed in detail. 

## 2. Experimental Section

### 2.1. Materials

Phenol, aniline, paraformaldehyde, chloroform, potassium hydroxide, absolute ethanol, and hydrochloric acid were purchased Tianjin Tianli Chemical Co., Ltd. (Tianjin, China). All the chemicals were used as received without any further purification. 

### 2.2. Preparation of PBZ-Based Nitrogen-Doped HPCs

Firstly, the benzoxazine monomer was prepared by a solventless method reported by Ishida [18]. Stoichiometric amount of phenol (39.0 g, 16.6 mol), aniline (61.2 g, 16.6 mmol), and paraformaldehyde (38.7 g, 33.2 mmol) were added into a 250-mL three-necked flask, and the mixture was refluxed and heated at 110–120 °C for 2 h and cooled down to room temperature. The crude product dissolved in chloroform was purified by 5% potassium hydroxide solution and distilled water repeatedly until a transparent yellow orange resin was obtained. Then, the obtained benzoxazine monomer was cured stepwise at 150, 180, 210, and 240 °C for 4 h to achieve the fully-polymerized polybenzoxazine. The cured PBZ was further carbonized at 500 °C for 2 h with an increasing rate of 5 °C min^−1^ under a nitrogen atmosphere, which was denoted as NPC-500. Subsequently, the NPC-500 were mixed with KOH (mass ratio of 1:1), and the mixtures were pyrolyzed at 600, 700 or 800 °C for 1 h with a ramp rate of 5 °C min^−1^ under a nitrogen atmosphere, respectively. Finally, the products were repeatedly washed with 1 M HCl solution and deionized water for several times and then dried at 120 °C for 12 h. The resulting carbon materials were denoted as HPC-600, HPC-700, and HPC-800, respectively.

### 2.3. Characterization

The microstructure of the product was observed with a JEOL S-4800 microscope (operating voltage = 10.0 kV). X-ray diffraction patterns of the samples were recorded by an X-ray diffractometer (Bruker D8, Cu Kα radiation, λ = 0.15406 nm). Raman spectra were recorded on a laser Raman spectrometer (Renishaw Invia) with an excitation wavelength of 532 nm. Nitrogen(N_2_) adsorption and desorption curves were measured using a Micromeritics ASAP2020 analyzer at −196 °C. The specific surface areas (S_BET_) were calculated by the Brunauer–Emmett–Teller (BET) method, and the surface area (S_micro_) and volume (V_micro_) of the micropores were estimated from the adsorption data by t-plotting. The total pore volume (V_total_) was calculated from the amount of adsorption when the relative pressure P/P_0_ was 0.99. The pore size distribution was also estimated from the adsorption branch of the isotherm using the Barrett–Joyner–Halenda (BJH) method. X-ray photoelectron spectroscopy (XPS) was performed using an AXIS Ultra DLD spectrometer with an excited MgKα source (1486.6 eV). Elemental analysis was obtained using an Elementar Vario Macro EL Cube microanalyzer. 

### 2.4. Electrochemical Measurements

The working electrodes were prepared by mixing 80 wt % the nitrogen-doped HPCs as an active material, 10 wt % acetylene black as a conductive agent and 10 wt % poly (tetrafluoroethylene) as a binder. The paste were pressed on a nickel foam current collector under a pressure of 8 MPa. The mass loading of the active material was in between 4 and 5 mg·cm^−2^. Electrochemical measurements were measured in a three-electrode system and a two-electrode system, respectively. In a three-electrode system, platinum sheet and saturated calomel electrode (SCE) were used as the counter electrode and the reference electrode, respectively. In a two-electrode system, the two electrodes of nearly the same mass and area were separated by a polypropylene film. Cyclic voltammetry (CV), galvanostatic charge/discharge (GCD), and electrochemical impedance spectroscopy (EIS) were measured in a 6 M KOH electrolyte with the electrochemical workstation CHI 660E instrument (Shanghai CHI Apparatus Co. Ltd., China). 

## 3. Results and Discussion

### 3.1. Structure and Morphology Characterization

**Figure 1:**
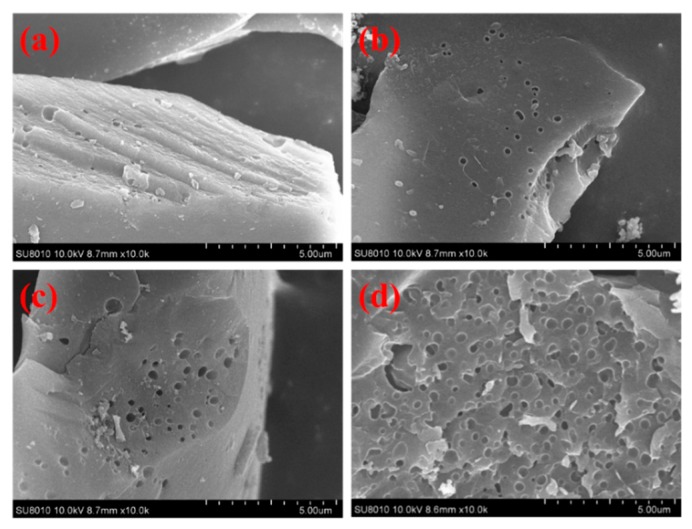
Scanning electron microscope (SEM) images of the NPC-500 (**a**); HPC-600 (**b**); HPC-700 (**c**); and HPC-800 (**d**).

The typical SEM images of the NPC-500 and HPCs materials are shown in Figure 1. The NPC-500 exhibits a rough surface with few macropores or cavities, which is considered to be beneficial for KOH chemical activation for developing the microporosity (Figure 1a). However, the HPC-600 performs many voids and significant macropores in 0.2-μm pore sizes, which results from the violent etching between carbon and KOH at high temperature (Figure 1b). As the KOH activation temperature is raised from 600 °C to 800 °C, more pores are formed and their diameter becomes larger. This causes the pore walls to become thinner and form more macroporous channels on the surface of the carbon skeleton, some of which are even in interconnect with each other. The abundant interconnected macropores and mesopores provide a channel for electrolyte transportation into the inner nanoporous carbon, by shortening molecular diffusion distance, and consequently enhancing their rate capability [19].

**Figure 2:**
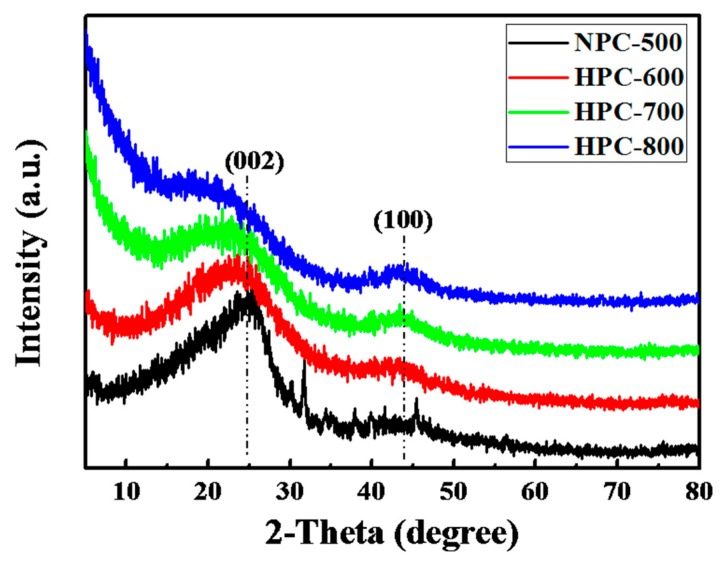
X-ray Diffraction (XRD) patterns of the NPC-500, HPC-600, HPC-700, and HPC-800.

The structure of the NPC-500 and HPCs materials was investigated by XRD. The XRD patterns of NPC-500 and HPCs are shown in Figure 2. The two broad diffraction peaks at approximately 2θ = 25° and 44° can be attributed to the (002) and (100) diffraction of the hexagonal graphite carbon lattice, respectively. Both the two peaks have a 2–3° left shift between the NPC-500 and the activated samples, which suggests the increscent lattice distance during the activation process. Besides, the (002) and (100) peak shapes are broadened, and their strength is gradually lowered as the activation temperature is raised from 600 °C to 800 °C, which indicates the formation of more amorphous and non-graphitized HPC. Moreover, the intensity of the diffraction peak of HPC-800 at low angle is significant, which means the existence of abundant pores [20]. 

**Figure 3:**
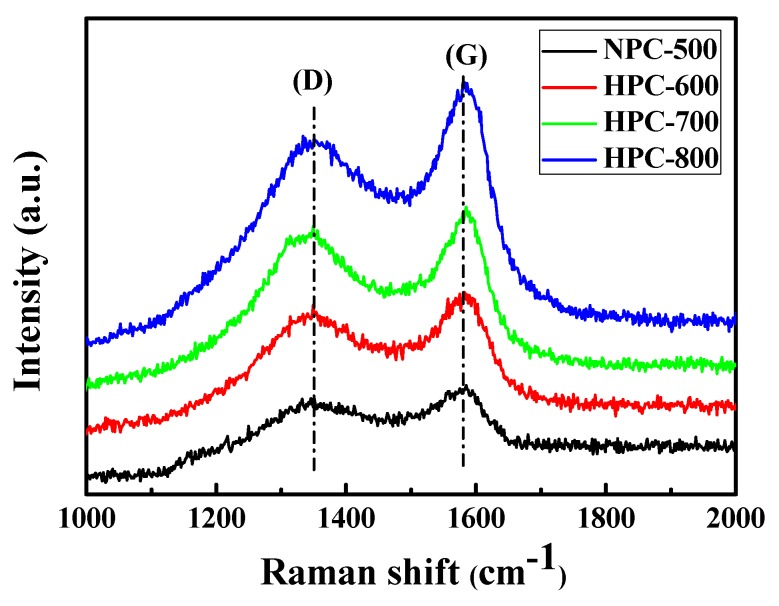
Raman spectra of the NPC-500, HPC-600, HPC-700, and HPC-800.

The graphitic structure of the NPC-500 and HPCs materials were further demonstrated by Raman spectroscopy, as shown in Figure 3. There are two strong Raman peaks at 1350 cm^−1^ (D band) and 1585 cm^−1^ (G band), respectively. The D band is assigned to the disordered and defective structures in the carbon material, while the G band is derived from the vibration of the sp^2^ carbon atoms in the graphite crystal [21]. The NPC-500 exhibits an intense D band and a weak G band, implying the presence of more defects, such as heteroatom doping. The graphite degree of the carbon material is represented by the intensity ratio of the D and G bands (I_D_/I_G_). The values of NPC-500, HPC-600, HPC-700, and HPC-800 are 1.11, 0.99, 0.90, and 0.84, respectively (listed in Table 1). This indicates that the disordered structure gradually increases and the degree of graphitization decreases with the increasing activation temperature, which is coincident with the XRD patterns. This can be assigned to the lower of nitrogen and oxygen sorts at higher activation temperature [22]. 

**Figure 4:**
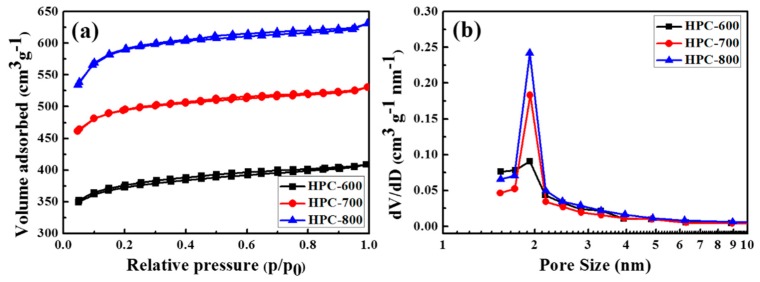
N_2_ adsorption isotherms (**a**) and Barrett–Joyner–Halenda (BJH) pore size distributions (**b**) for the HPCs.

The specific surface area and porous structure of the HPCs were characterized by N_2_ adsorption-desorption technique. In Figure 4a, all the HPCs exhibit typical IV isotherms with a large hysteresis loops at relative pressures p/p_0_ > 0.5, suggesting the generation of massive mesopores. At low relative pressures p/p_0_ < 0.1, the isotherms have certain vertical displacement increase, indicating the presence of limited micropores. At high relative pressure p/p_0_ > 0.9, an obvious rise of the isotherms can be attributed to the presence of few macropores, which is in agreement with the results of the SEM images [23]. That is to say, all the HPCs exhibited hierarchical porous structures: limited micropores, abundant mesopores, and few macropores. Table 1 exhibits details of the textual characteristics of the HPCs. The Brunauer–Emmett–Teller (BET) surface area of HPC-600, HPC-700, and HPC-800 are 1136.6, 1506.5, and 1812.4 cm^2^·g^−1^, respectively. The KOH activation temperature had an outstanding impact on developing the micropore structure [24]. As the KOH activation temperature was raised from 600 to 800 °C, new micropores emerged and the ultra-micropores were enlarged, leading to higher surface area and large total pore volume (see Table 1). The total pore volumes of HPC-600, HPC-700, and HPC-800 were 0.63, 0.82, and 0.98 cm^3^·g^−1^, respectively. The pore size distribution of the HPCs was further demonstrated by the BJH method, as shown in Figure 4b. As the KOH activation temperature increases, the average pore diameter changes from 1.89 to 1.92 nm. This indicates that the mesopores were widened and micropores were grown by KOH etching under harsh activation temperature [25]. 

**Figure 5:**
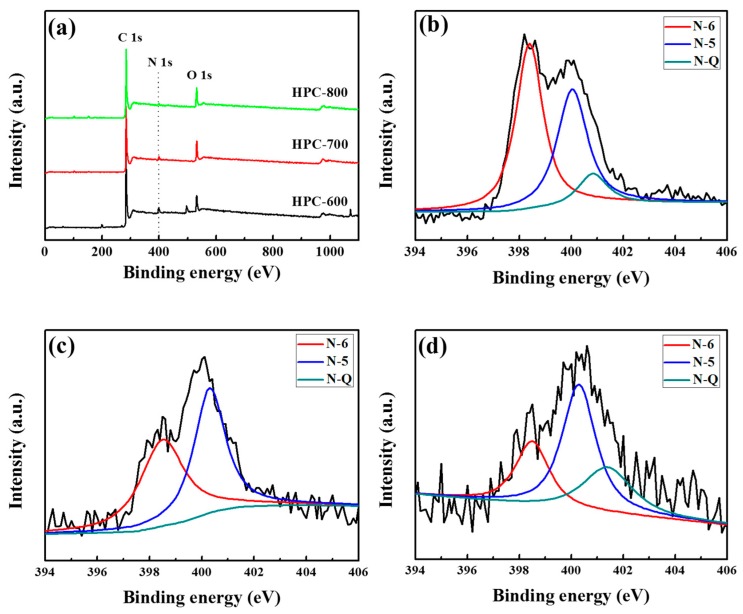
X-ray photoelectron spectroscopy (XPS) survey spectra (**a**); N 1s spectra of the HPC-600 (**b**); HPC-700 (**c**); and HPC-800 (**d**).

The chemical compositions of the HPCs were investigated by Elemental analysis. The HPC materials possess relatively high nitrogen species (1.27–3.45%), which can be obtained from the intrinsic nitrogen component in the PBZ (see Table 1). The properties of the nitrogen functional groups on the surface of the HPC carbon material analyzed by XPS are shown in Figure 5. Three most pronounced styles of nitrogen species can be discriminated on the N 1s spectrum: quaternary N (N-Q at 401.4 eV), pyrrolic or pyridonic N (N-5 at 400.3 eV), and pyridinic N (N-6 at 398.5 eV), respectively [26]. It can be found that N-5 and N-6 species are critical in the HPCs, which might result from the conversion of amide groups in the PBZ after heat treatment. With an increase of the activation temperature, N-6 content were gradually decreased, but more N-5 and N-Q species formed for the HPCs, which indicates that N-6 were partially transformed to N-5 and N-Q at higher temperature. It has been reported that N-6 and N-5 exhibit much higher electrochemical activity in supercapacitors to offer more pseudo-capacitance, which is important for nitrogen-doped porous carbons to improve the specific capacitance [27]. Furthermore, the existence of N-Q, which are incorporated into the carbon network and bonded to three carbon atoms, can enhance electron transfer and electrical conductivity [28]. Therefore, it can be concluded that the HPC-800 can generate primary pseudo-capacitance in an alkaline aqueous electrolyte due to the high N-Q content and high electrical conductivity.

### 3.2. Electrochemical Measurements

**Figure 6:**
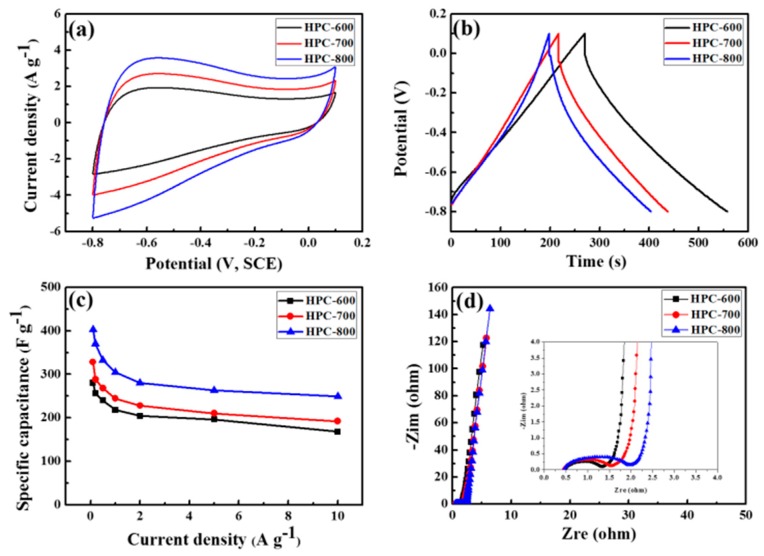
Electrochemical performance of all the HPC electrodes measured in a three-electrode system: (**a**) Cyclic voltammetry (CV) curves at a scan rate of 10 mV·s^−1^; (**b**) Galvanostatic charge/discharge (GCD) curves at a current density of 1 A·g^−1^; (**c**) specific capacitances at different current densities; and (**d**) Nyquist plots and inset magnified image.

The electrochemical properties of all the HPC electrodes for supercapacitors was carried out in a 6 M KOH electrolyte via a three-electrode system. Figure 6a exhibits the CV curves of all the HPC electrodes at a sweeping rate of 10 mVs^−1^. All the electrodes exhibit typical quasi-rectangular shapes with small, broad redox peaks, suggesting the existence of pseudo-capacitance arising from Faradaic reactions of the N and O heteroatoms in the carbon network. The HPC-800 exhibits the largest rectangular-like shape according to the highest specific capacitance among the three HCP materials, which can be ascribed to its highest specific surface area and largest pore volume. Figure 6b shows the GCD profiles of the HPC electrodes at a current density of 1.0 A·g^−1^. The HPC-800 electrode exhibits a nearly symmetric triangular shape with slight curvature and the largest discharge time. It suggests that the HPC-800 electrode possesses the largest specific capacitance, which confirms the aforementioned CV results. Figure 6c shows the specific capacitances at various current densities of all the HPC electrodes. The capacitance of all the electrodes decreases slowly with increasing current density, which indicates that it has fast charge and discharge characteristics and good rate performance. Notably, the HPC-800 electrode exhibits the largest capacitance in comparison with the other two electrodes. The specific capacitance of the HPC-800 electrode at 0.1, 1.0, and 10 A·g^−1^ preserves very high capacitances of 402.4, 304.3, and 248.6 F·g^−1^, respectively, which is equivalent to the capacitance retention of 100%, 75.6%, and 61.8% of the value at 0.1 A·g^−1^. The HPC-800 possesses high specific capacitance and good rate performance because of its high surface area, large pore volume, optimal pore size distribution, abundant micro/mesoporous structure, large amount of electrochemically, and active N and O species in the carbon network. Figure 6d shows the Nyquist plots of the HPC electrodes after GCD measurement, where the Zre reflects the equivalent ohmic resistance and the Zim corresponds to the existence of non-resistive atoms. Nyquist plots comprise a semicircle, a linear part with a slope of 45º and a nearly vertical line. The intercept at the Zre impedance axis is related to the internal resistance of the electrode. The internal resistances of HPC-600, HPC-700, and HPC-800 electrodes are 0.49, 0.45, and 0.44 Ω, respectively, indicating that the HPC-800 electrode possesses the best electrical conductivity. In the high frequency area, a larger diameter of the semicircle for the electrode reflects the existence of higher Rct (charge-transfer resistance), suggesting fast charge transfer in the hierarchical porous structure of the HPC-600 electrode [29]. In the medium frequency area, with the increase of activation temperature, the slope line of the HPC-800 electrode obviously becomes shortened, indicating the lowest Warburg impedance diffusion resistance, presumably because of its widened pore size. In the low-frequency area, the oblique lines of the HPC electrodes are close to the theoretical vertical line and exhibit typical features of pure capacitive behavior. These results confirm that the hierarchical pore structure with plays an important role in high-rate supercapacitors [30].

**Figure 7:**
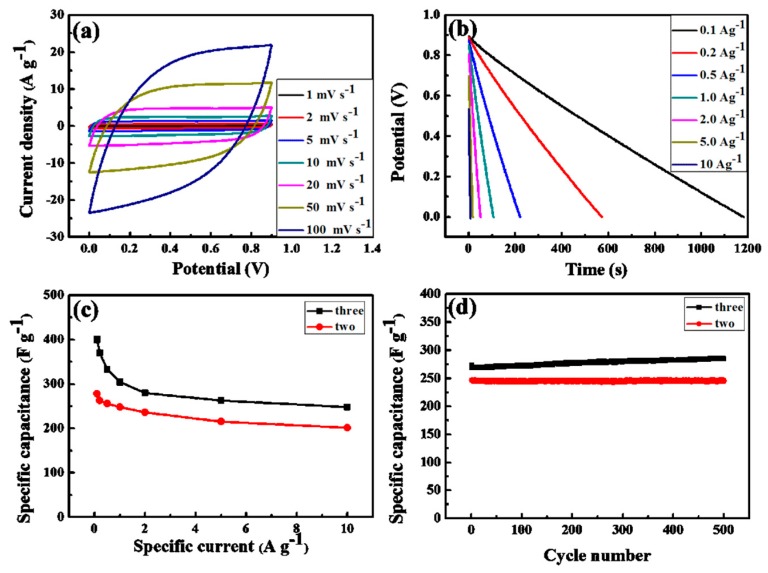
Electrochemical performance of the HPC-800 electrode measured in a two-electrode system: (**a**) CV curves at different various scan rates; (**b**) GCD curves under different current densities; (**c**) capacity profiles at different current densities; (**d**) the long cycle life over 500 cycles at a current density of 1 A·g^−1^.

A symmetric two-electrode supercapacitor in a 6 M KOH aqueous electrolyte was used to measure the electrochemical behavior of the HPC-800 electrode. Figure 7a is the CV curves of the HPC-800 electrode at various sweeping rates. It can be found that the current densities increase obviously with an increase of the sweeping rates, and a similar rectangular shape is retained, implying the good rate capacity, which may arise from the high porosity of HPC-800 allowing a fast transmission of ions in the carbon framework. The galvanostatic discharge curves at various current densities are shown in Figure 7b. The discharge time decreases accordingly with the current densities increase from 0.1 to 10 A·g^−1^, and no obvious changes of shape happened, which agree with the aforementioned CV results. In general, the specific capacitance of a single HPC-800 electrode can be calculated from the equation of *C* = 4IΔ*t*/(mΔV). The specific capacitances for a single HPC-800 electrode by using a two-electrode system are 278.2, 263.5, 256.7, 248.4, 236.3, 215.1, and 202.8 F·g^−1^ at 0.1, 0.2, 0.5, 1, 2, 5, and 10 A·g^−1^, respectively, which are slightly smaller than the values in a three-electrode system due to the different measurement methods, as shown in Figure 7c. It also indicates an excellent rate stability with a good capacitance retention ratio of 72.9% at 10 A·g^−1^. Figure 7d shows the cycling stability was investigated at a current density of 1 A·g^−1^. During the cycle time, the surface of carbon electrode was wetted well by electrolyte-ions [31]. After 500 cycles, the specific capacitance displays a capacitance retention ratio of greater than 99.0%, implying a good cycle stability of the nitrogen-doped carbons. These results indicated that the HPC-800 electrode possesses excellent electrochemical capacitive performance because of their high specific surface area, optimal hierarchical pore structure, and electrochemically active nitrogen functional groups.

## 4. Conclusions

Novel nitrogen-doped hierarchically porous carbons derived from PBZs were successfully prepared through the KOH chemical activation method. The KOH chemical activation temperature had a significant effect on the structural properties and the surface chemistry of HPCs, which will further affect their capacitive behaviors. The HPC-800 electrode exhibited the highest specific capacitance of 402.4 F·g^−1^ at 0.1 A·g^−1^ and 248.6 F·g^−1^ at 10 A·g^−1^ in a three-electrode system. Moreover, the HPC-800 electrode still retained a capacitance retention ratio of greater than 99.0% after 500 charge/discharge cycles. The excellent electrochemical performance of the HPC-800 is attributed to its properties, such as high specific surface area, optimal pore size distribution, and abundant nitrogen functionality. This is a promising alternative for high-performance electrode materials in supercapacitors.

## Figures and Tables

**Table 1:** Textural Properties of the HPCs.

Items	I_D_/I_G_	S_BET_ (cm^2^·g^−1^)	V_total_ (cm^3^·g^−1^)	V_micro_ (cm^3^·g^−1^)	Pore Radius (nm)	N (wt %)
HPC-600	0.99	1136.6	0.63	0.54	1.89	3.45
HPC-700	0.90	1506.5	0.82	0.73	1.91	2.42
HPC-800	0.81	1812.4	0.98	0.88	1.92	1.27
